# Extra Fees

**Published:** 1875-12

**Authors:** 


Extra Fees.—With a view to encourage patients and others, who may be in need of the attendance of a physician, to send him word at such time as will admit of his arranging his work for the day, the Forfarshire (Scotland) Medical Association have confirmed the resolution unanimously adopted at last year's meeting, "that all visits sent for after 10 a.m., and requiring to be attended to the same day, should be charged at an extra rate."—*New York Medical Advertiser.*

